# Tri‐Layer Solid‐State Nanopore Arrays with Crosstalk Suppression for High‐Throughput, Femtomolar‐Level Biosensing

**DOI:** 10.1002/advs.74213

**Published:** 2026-02-04

**Authors:** Silu Feng, Qinglong Luo, Siqi Ai, Suiwei Shen, Chengyong Wang, Zhishan Yuan

**Affiliations:** ^1^ State Key Laboratory of High‐Performance Tools Guangdong University of Technology Guangzhou China; ^2^ School of Integrated Circuits Guangdong University of Technology Guangzhou China; ^3^ School of Electromechanical Engineering Guangdong University of Technology Guangzhou China; ^4^ Guangdong Provincial Key Laboratory of Minimally Invasive Surgical Instruments and Manufacturing Technology Guangdong University of Technology Guangzhou China

**Keywords:** Al_2_O_3_/Au/Si_3_N_4_ sandwich structure, high‐throughput molecular detection, scalable nanofabrication, solid‐state nanopore arrays

## Abstract

Solid‐state nanopore arrays are emerging as powerful tools for label‐free, ultrasensitive biosensing, yet their implementation has been constrained by inter‐pore crosstalk and limited fabrication uniformity. A multilayer Al_2_O_3_/Au/Si_3_N_4_ nanopore architecture, produced via helium ion beam lithography, is introduced to address these limitations through structural and materials‐level innovation. Finite‐element analysis identifies a critical inter‐pore spacing approximately 20 times the pore radius as necessary to minimize electric field coupling, enabling rational array design. The membrane structure incorporates a dielectric Al_2_O_3_ layer for electrical isolation and an intermediate gold layer for site‐specific aptamer immobilization, confining molecular recognition to the nanopore interior. Arrays with ∼30 nm pores and <5% size variation achieve 300 nm spacing and support statistically independent, parallel signal acquisition. Diverse nanopore arrays with 75 nm pores and 800 nm spacing are utilized for the specific detection of alpha‐fetoprotein. Detection of alpha‐fetoprotein demonstrates label‐free sensing at concentrations down to ∼3 fM across six orders of magnitude in dynamic range. This platform defines a closed‐loop pathway from theoretical modeling to scalable fabrication, establishing a foundation for rational design and high‐throughput deployment of solid‐state nanopore biosensors.

## Graphical abstract

The critical pore spacing—approximately twenty times the pore radius—required to minimize electric field coupling is determined via finite element analysis. A multi‐layered Al_2_O_3_/Au/Si_3_N_4_ nanopore structure fabricated by helium ion beam lithography is proposed, enabling quantitative analysis of the target analyte while mitigating inter‐pore crosstalk.

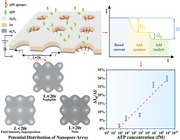

## 1 Introduction

Solid‐state nanopore arrays, featuring precisely tunable dimensions, label‐free detection capabilities, and high sensitivity, have become essential elements in cutting‐edge biosensing technologies. These arrays are particularly influential in single‐molecule detection across a variety of applications, including DNA sequencing [1, 2, 3], protein analysis [4, 5], environmental monitoring [6, 7], and bionic system construction [8, 9]. By allowing multiple nanopores to operate as statistically independent sensing units, the array format dramatically increases the effective sampling volume and event frequency, mitigating Poisson‐limited fluctuations at femtomolar analyte concentrations and enabling multiplexed, high‐throughput molecular detection. When nanopores are positioned too closely, the overlapping of electric fields and diffusion layers induces inter‐pore crosstalk, which compromises detection fidelity and quantitative accuracy [10].

Reducing molecular translocation rates can partially alleviate this interference, but it inevitably decreases throughput and conflicts with the intrinsic advantage of array‐based platforms [11]. Recent theoretical analyses have predicted critical spacing thresholds by modeling coupled electrical and diffusive interactions between adjacent nanopores, suggesting that an inter‐pore distance several 10 of times the pore diameter is required to suppress cross‐coupling [12, 13, 14]. While these predictions provide valuable design guidance, systematic experimental validation remains lacking. Most reported nanopore arrays therefore adopt conservative geometries with limited nanopore counts [15, 16] or excessively large spacing [17, 18], leaving the quantitative relationship between pore spacing, signal independence, and detection performance largely unverified. This absence of experimental evidence has hindered the rational design of high‐density nanopore arrays capable of achieving both ultrasensitivity and true high‐throughput operation.

Large‐area nanopore arrays with uniform structure are essential for scalable biosensing, yet remain difficult to fabricate. Conventional Ga^+^ FIB drilling is limited by beam fluctuations and inconsistent material responses, causing pore‐size variations across arrays [19]. Helium ion microscopy (HIM) offers higher spatial resolution and etching precision with reduced membrane damage, enabling fabrication of high‐density arrays with improved uniformity [20]. However, high‐energy He^+^irradiation can still induce subsurface amorphization and ion implantation, affecting long‐term device stability. To overcome these trade‐offs, a multilayer “sandwich” architecture is introduced, integrating dielectric, conductive, and structural layers to achieve reproducible patterning, mechanical robustness, and electrical isolation in a single platform.

In this study, finite‐element simulations of ionic diffusion and an delectric‐field ovrelap were employed to establish the design parameters required for independent operation of adjacent nanopores. The modeling indicated that an inter‐pore distance of approximately 20 times the nanopore diameter effectively suppresses field coupling and minimizes signal interference. To satisfy these constraints while maintaining mechanical integrity and chemical addressability, a multiplayer Al_2_O_3_/Au/Si_3_N_4_ sandwich‐structured nanopore array was developed. In this configuration, the Al_2_O_3_ dielectric layer minimizes electric‐field leakage through the metallic film, and the intermediate Au layer serves as a functional interface for thiolated aptamer immobilization via Au─S bonding. The differential binding affinities among these layers confine aptamer functionalization within the nanopore interior, effectively eliminating nonspecific adsorption on the membrane surface and improving the reproducibility of molecular recognition. The array was fabricated using HIM, which produced uniform nanopores with an average diameter of about 30 nm and a size variation below 5%. The resulting layout, featuring a 300 nm inter‐pore distance consistent with theoretical predictions, enables statistically independent and parallel current acquisition without requiring high bias voltages that could destabilize aptamer and target complexes. Using alpha‐fetoprotein (AFP) as a representative biomarker, the array achieved a detection limit of ∼3fM across 6 orders of magnitude in dynamic range, with an apparent dissociation constant (K_d_) of 0.34 nm, demonstrating accurate sub‐nanomolar quantification.

In summary, this study presents a unified theoretical–experimental framework that bridges nanoscale modeling with experimental realization. The multilayer Al_2_O_3_/Au/Si_3_N_4_ nanopore array achieves high‐fidelity, label‐free, and quantitative biosensing with ultrahigh sensitivity and scalability. By integrating validated spacing theory, structural innovation, and complete biomolecular demonstration, this work not only advances the rational design of solid‐state nanopore arrays but also establishes a versatile platform for next‐generation analytical technologies. Its modular and generalizable architecture provides broad applicability across biomedical diagnostics, environmental pollutant monitoring, and precision healthcare, highlighting the potential of solid‐state nanopores as foundational tools for intelligent sensing and molecular analytics.

## 2 Results and Discussion

### 2.1 Design and Fabrication of Nanopore Arrays

#### 2.1.1 Tumor Marker Detection Principle of Nanopore Arrays

**FIGURE 1:**
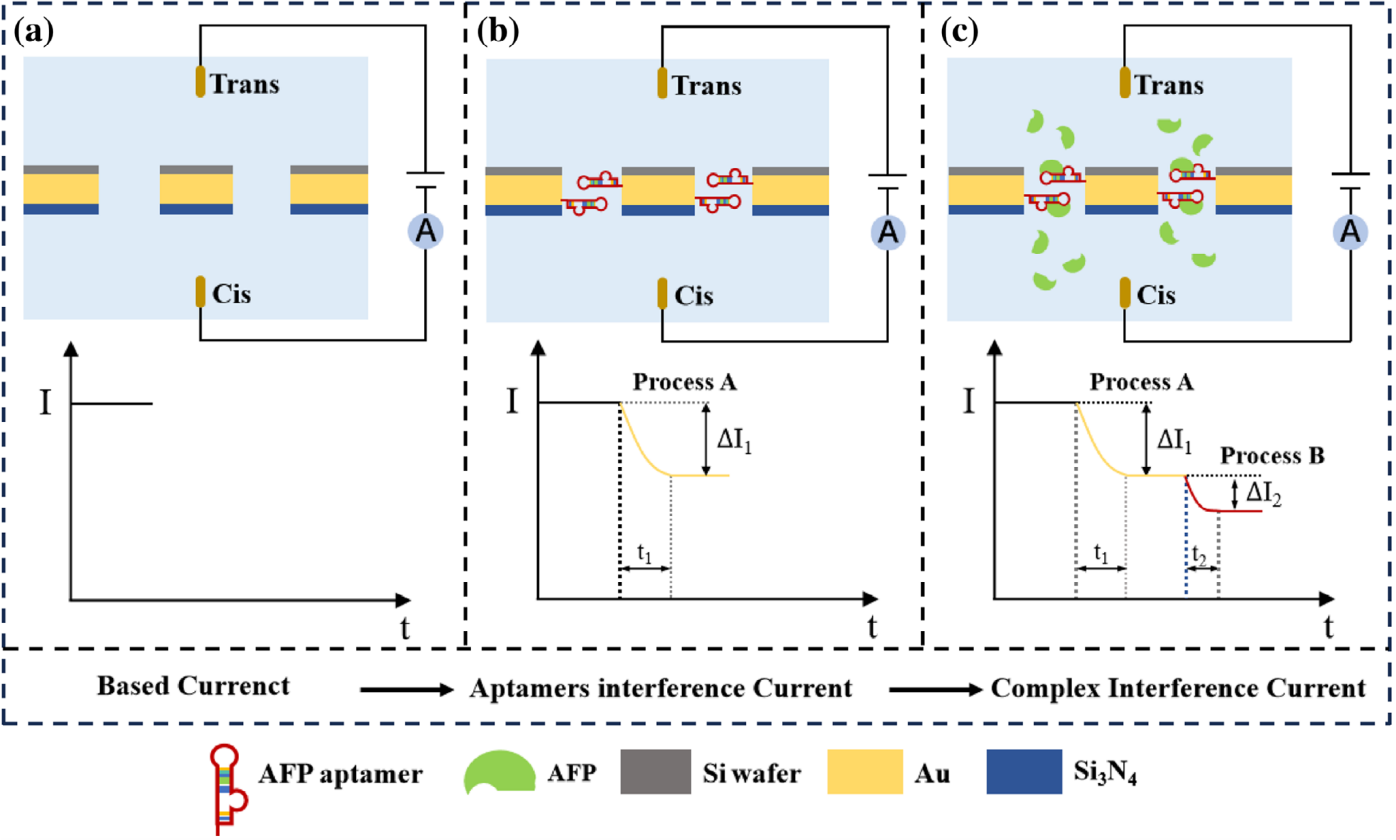
Tumor marker detection principle of Al_2_O_3_/Au/Si_3_N_4_ nanopores array sandwich. (a) Recording the initial current baseline; (b) Adding aptamer (red) and immobilizing it on the gold layer, followed by determining the current decrease amplitude (ΔI_1_) induced by the aptamer modification; (c) Adding AFP antigen, which binds to the immobilized aptamer, and acquiring the current decrease amplitude resulting from this binding event (ΔI_2_).

The AFP detection principle based on Al_2_O_3_/Au/Si_3_N_4_ sandwich nanopore arrays is illustrated in Figure 1. The Al_2_O_3_/Au/Si_3_N_4_ sandwich nanopore array was installed between two electrolyte‐filled chambers, with an external voltage applied via Ag/AgCl electrodes. The current of the nanopore array was then recorded, as shown in Figure 1a. Subsequently, the nanopore array was removed and chemically modified by attaching AFP aptamers to the gold surface of the nanopores. The current was measured again after the nanopore walls were modified with AFP aptamers, and the current difference between the modified and unmodified nanopores was calculated as ΔI_1_, as depicted in Figure 1b. Finally, AFP antigen at varying concentrations was introduced into the Cis chamber. Under the influence of an electric field, the AFP antigen entered the nanopores and was captured by the AFP aptamers, resulting in a decrease in the baseline current. The difference between the baseline current and the current through the nanopores with AFP aptamers on the surface was recorded as ΔI_2_, as shown in Figure 1c. By comparing the proportion of the current difference ΔI_2_ to the total current change ΔI_1_+ ΔI_2_. The concentration of the AFP antigen can be determined, enabling quantitative detection of liver cancer tumor markers.

#### 2.1.2 Design and Simulation of Inter‐Pore Distance in Nanopore Arrays

Solid‐state nanopore arrays represent a pivotal strategy for achieving high‐throughput, high‐efficiency single‐molecule detection, where inter‐pore distance emerges as a critical parameter governing sensing performance. Adequate spacing between nanopores ensures that each nanopore operates independently, maintaining high current fidelity and quantitative precision. Conversely, insufficient spacing leads to overlapping electric fields and analyte competition, which compromise both throughput and accuracy [21]. Although theoretical studies consistently emphasize that sufficient separation suppresses inter‐pore crosstalk, a quantitative consensus on the critical spacing threshold has not yet been established [22, 23].

**FIGURE 2:**
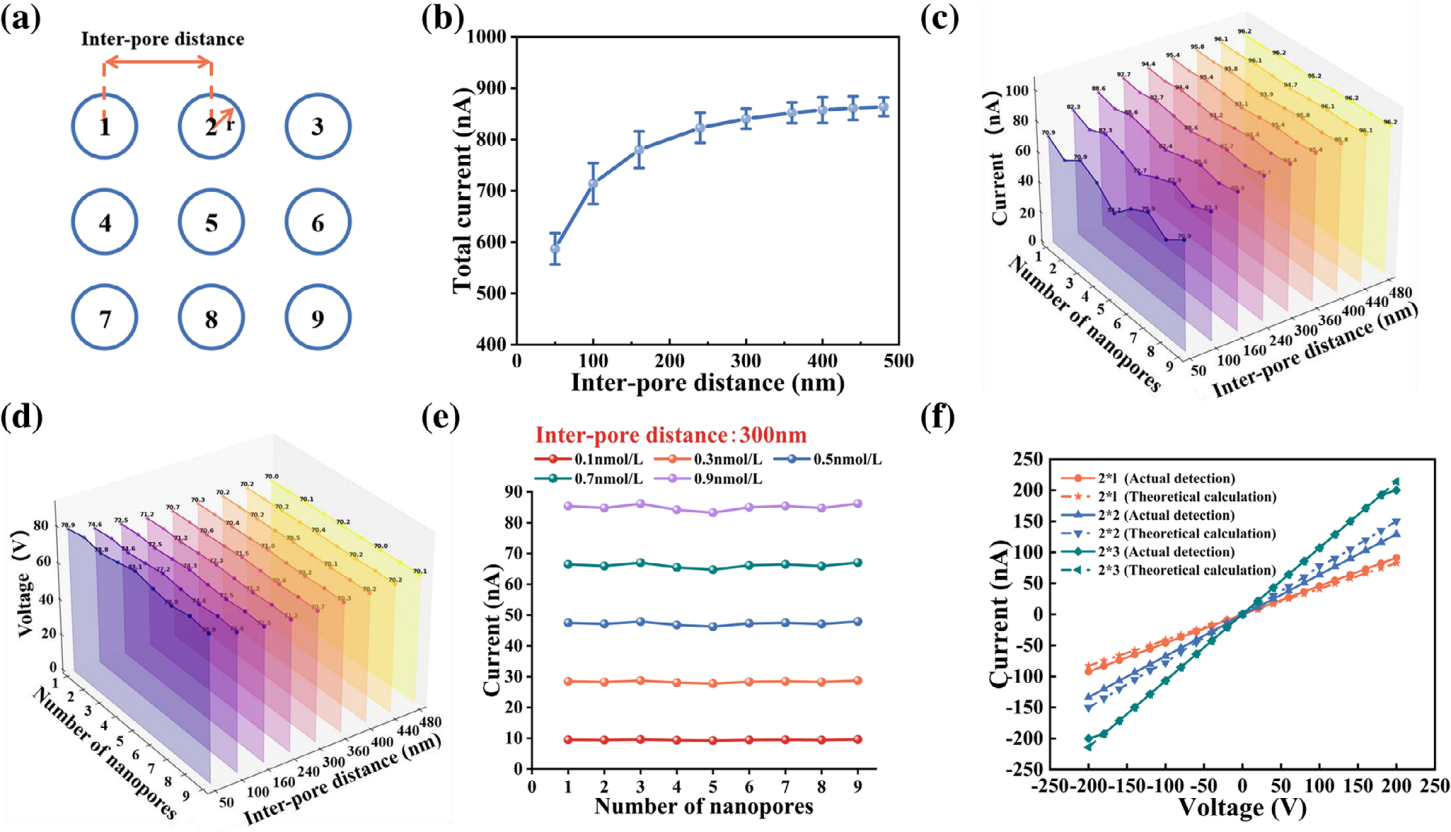
Simulation of inter‐pore distance in nanopore arrays based on COMSOL. (a) Schematic diagram of 3 × 3 array nanopore layout; (b) Total current of 3 × 3 nanopore array as a function of inter‐pore distance; (c) 3D waterfall diagram of current and inter‐pore distance at different positions in 3 × 3 nanopore array. (d) 3D waterfall diagram of potential and inter‐pore distance at different positions in a 3 × 3 nanopore array. (e) The function of current with electrolyte concentration and nanopore position when the inter‐pore distance is 300 nm; (f) Comparison of *I–V* curves obtained by theoretical calculation and actual detection.

Herein, finite element simulations were performed using COMSOL Multiphysics 5.6 to systematically investigate the effect of inter‐pore distance on the ionic current characteristics of a 3 × 3 nanopore array. The geometric configuration of the nanopore array is illustrated in Figure 2a, with the detailed ionic current calculation formulas, COMSOL model setup, and parameter specifications provided in T1 and T2 of the Supporting Information. The nanopore radius was fixed at 15 nm, and the ratio of inter‐pore distance to pore radius (L/r) was used as the metric to find the threshold for inter‐pore distance. (detailed parameters are provided in Table S2). As illustrated in Figure 2b, the total ionic current of the array exhibits a non‐linear increase with increasing inter‐pore distance, and tends to saturation when the inter‐pore distance reaches ∼300 nm (L/r = 20). This saturation behavior is governed by ion concentration polarization (ICP), a characteristic effect in nanofluidic transport that produces ion depletion and enrichment zones near charged nanopore orifices [24, 25, 26].

At the pore entrance, negatively charged pore walls attract counterions and repel co‐ions, forming an ion depletion zone (IDZ) at the pore entrance and an ion enrichment zone (IEZ) at the exit. The superposition of the surface‐charge‐induced field and the applied bias enhances ion mobility within the IDZ, creating an imbalance between ionic migration and diffusion. When adjacent nanopores are closely spaced, overlapping IDZs hinder ion replenishment from the bulk solution and suppress the ionic current [27, 28]. As the inter‐pore distance increases, the overlap between neighboring IDZs and IEZs gradually diminishes and eventually disappears, allowing each nanopore to operate independently.

3D multi‐section processing was performed on the simulation output data, with data and range regulated to obtain the electric potential distribution maps of the 3 × 3 nanopore array in the xy‐plane and yz‐plane (Figures S1 and S2). Integration of the aforementioned potential data and ionic current data enables systematic investigation of the influence of nanopore position within the 3 × 3 array on ionic current and electric potential under various spacing conditions (Figure 2c,d). Overall, the ionic current increased with inter‐pore distance, consistent with the trend in Figure 2b, whereas the electric potential showed an inverse behavior and reached a plateau once the spacing exceeded the threshold. At reduced spacing (e.g., 50 nm), a clear positional dependence was observed: corner pores exhibited the highest current and lowest potential, edge pores showed intermediate values, and the central pore displayed the lowest current and highest potential. This spatial variation reflects differences in IDZ overlap and electric‐field superposition, which are most pronounced when the pores are closely spaced.

At subcritical pore spacing, lateral electric‐field components generated by neighboring nanopores weaken the axial electrophoretic force within the molecular capture region, effectively reducing the capture radius and resulting in more tortuous translocation trajectories. These interactions broaden molecular residence‐time distributions, lower event frequency, and increase the variability of blockade amplitudes and durations, thereby degrading the signal‐to‐noise ratio and detection accuracy. The ionic current also scales with electrolyte concentration, increasing with elevated ionic strength (Figure 2e). The influence of electrolyte concentration under other inter‐pore spacing conditions is illustrated in Figure S3. However, at small inter‐pore distances, high ionic strength intensifies current suppression due to enhanced inter‐ionic repulsion, which decreases ion mobility and exacerbates the migration–diffusion imbalance within overlapping IDZs. Taken together, these results indicate that an optimal spacing ratio of L/r = 20 ensures independent nanopore operation, establishing a theoretical foundation for subsequent array fabrication and experimental validation.

#### 2.1.3 Fabrication of Al_2_O_3_/Au/Si_3_N_4_ Nanopore Array Sensor

Schematics of the Al_2_O_3_/Au/Si_3_N_4_ nanopore array sensor fabrication are illustrated in Figure S4. The Si wafer underwent low‐pressure chemical vapor deposition (LPCVD) to sequentially deposit 20 nm of Si_3_N_4_, 300 nm of SiO_2_, and another 100 nm of Si_3_N_4_ (low stress). Reactive ion etching (RIE) was subsequently applied to pattern and etch the back of the wafer, followed by the etching of the Si_3_N_4_, SiO_2_, and Si_3_N_4_ layers to release the Si substrate. To create a suspended structure, a potassium hydroxide (KOH) etching process was employed at 85°C for 5.5 h, resulting in a 300 µm × 300 µm square‐shaped Si_3_N_4_/SiO_2_/Si_3_N_4_ composite membrane. Photolithography and reactive ion etching were then used to pattern and etch a 3.2 µm × 3.2 µm circular window on the front side of the wafer, exposing the sacrificial SiO_2_ layer. The SiO_2_ sacrificial layer was removed through a buffered oxide etch (BOE), leaving behind a 3.2 µm × 3.2 µm circular suspended Si_3_N_4_ membrane. Subsequently, a 50 nm thick TiW/Au layer was deposited on the front side of the wafer using electron beam evaporation. Atomic layer deposition (ALD) was then used to deposit a 20 nm thick Al_2_O_3_ layer on top of the TiW/Au surface. Finally, the wafer was diced into 3.5 mm × 3.5 mm individual Al_2_O_3_/Au/Si_3_N_4_ sandwich membrane chips.

The Al_2_O_3_/Au/Si_3_N_4_ Sandwich membrane chips were immersed in a piranha solution ($V_{H_{2}SO_{4}}$: $V_{H_{2}O_{2}}$= 7:3) and subsequently placed into a 90°C water bath for 30 min to remove surface impurities. The chips were then transferred into a 20 mL ethanol solution and soaked for 20 min to eliminate any remaining piranha solution residues from the surface. Following this, the chips were dried in a vacuum oven at 60°C for 6 h.

#### 2.1.4 Effect of Helium Ion Dose on Nanopore Diameter

The influence of HIM parameters on nanopore morphology was investigated through a series of fabrication experiments on Al_2_O_3_/Au/Si_3_N_4_ sandwich membranes using a circular beam pattern with a preset diameter of 50 nm. The acceleration voltage was fixed at 30 kV, and the chamber pressure was maintained at 2 × 10^−6^ Torr to ensure consistency. Ion currents of 20 pA, 15 pA, and 10 pA were applied, each with a range of ion doses (detailed in Table S3), while all other parameters were held constant. Representative HIM images are shown in Figure S5a–c. Although circular morphology was largely retained, slight asymmetries appeared due to beam focusing limitations. Pore dimensions were quantified by measuring the projected area with Image‐Pro and calculating equivalent diameters assuming perfect circularity (Figure S5d).

At constant ion current, pore diameter increased with He^+^ dose, but the rate of growth slowed due to the Gaussian beam profile. High‐energy ions in the beam core dominate sputtering, whereas peripheral ions contribute less once the pore exceeds the beam's high‐intensity center. At a fixed dose, increasing the ion current also resulted in larger pores, due to broader beam spot sizes enhancing lateral etching. Despite differences in absolute size, a consistent diameter–dose relationship was observed across all current levels.

#### 2.1.5 Experimental Validation of Nanopore Inter‐Pore Distance

Building on the simulation findings that established a minimum inter‐pore distance of 300 nm to avoid electro‐diffusion interference, nanopore arrays with 2 × 1, 2 × 2, and 2 × 3 configurations were fabricated accordingly. Each array maintained an inter‐pore distance of 300 nm and a nanopore diameter below 30 nm to ensure independent ion transport. The experiments were performed in a 1 mol/L KCl electrolyte solution.

To validate the theoretical model, ion currents were calculated using the formula in T1 (Equation S7) under the assumption of independent nanopore behavior, whereby the total current equals the product of single‐pore current and the number of pores in the array. The corresponding *I–V* curves were then compared with experimental data (Figure 2f), showing strong agreement and confirming the simulation‐based design strategy. A minor deviation in the 2 × 2 array suggests partial pore blockage during measurement. In contrast to single‐pore systems—where complete obstruction halts ion flow—array systems maintain current due to parallel conduction pathways. Therefore, discrepancies between theoretical and measured values serve as indirect indicators of pore‐level obstruction and offer a practical means to evaluate array integrity during sensing operations.

### 2.2 Influence of Different Parameters on Blocking Current Signal

Unless otherwise specified, the experimental parameters were set to the following defaults: a 2 × 2 sandwich‐structured nanopore array sensor with a pore diameter of 75 nm, an AFP concentration of 10 fm, 0.1 m KCl solution as the electrolyte, and an applied voltage of 200 mV.

#### 2.2.1 Electrolyte Concentration Gradient

To evaluate the influence of electrolyte concentration gradient on the sensing performance of nanopore arrays, experiments were conducted under transmembrane concentration gradient conditions in this study. The experimental device diagram can be seen in Figure S6. The electrolyte concentration on the cis side was maintained at 0.1 mol/L, while the concentration on the trans side was adjusted to 0.1 mol/L, 0.4 mol/L, 0.6 mol/L, and 1.0 mol/L, corresponding to concentration ratios (trans/cis) of 1, 4, 6, and 10, respectively.

**FIGURE 3:**
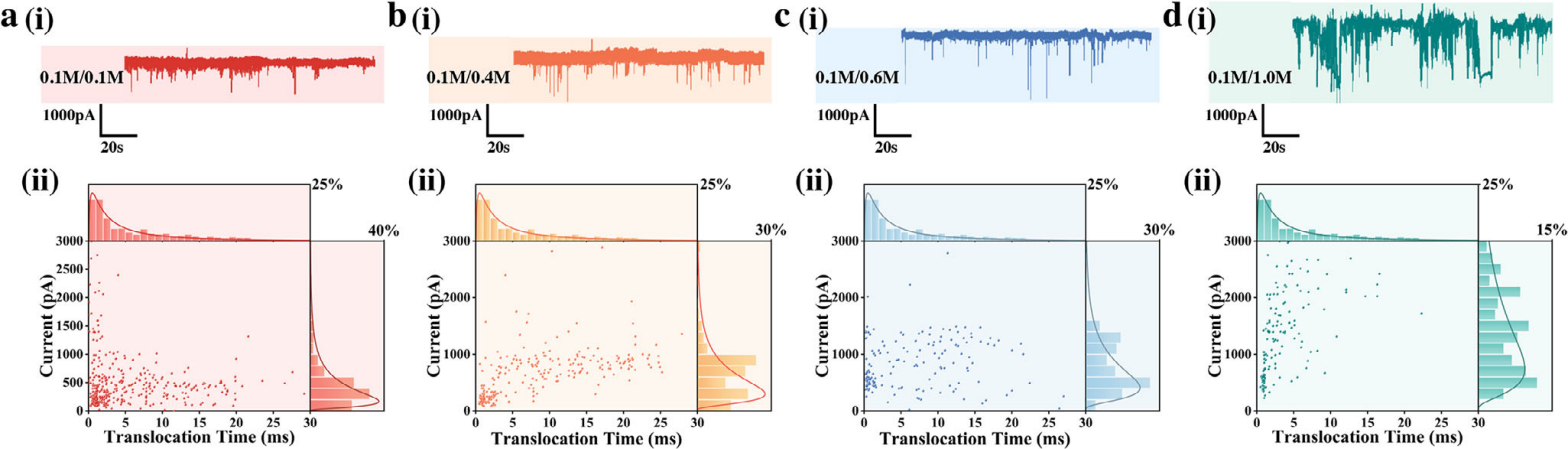
The influence of electrolyte concentration on current and translocation time. (a) 0.1 m/0.1 m, (b) 0.1 m/0.4 m, (c) 0.1 m/0.6 m, (d) 0.1 m/1.0 m. (i) Typical current trace; (ii) Scatter diagram of event distribution and normalized fitting Gaussian distribution diagram.

As shown in Figure 3i, increasing the electrolyte concentration gradient led to enhanced both the frequency and amplitude of current blockade events associated with AFP translocation. However, this improvement was accompanied by a concurrent rise in baseline noise and signal fluctuation. At a trans‐side concentration of 1.0 m, pronounced baseline oscillations and elevated noise levels were observed, which hindered accurate discrimination of translocation signals and reflected reduced signal stability under high concentration gradients. This phenomenon can be attributed to intensified ICP at large concentration differences across the nanopore array. The boundaries of the IDZ and IEZ fluctuate dynamically due to stochastic ion diffusion, thereby amplifying the background noise [29, 30, 31].

The effect of the concentration gradient was further analyzed by statistically evaluating the current blockade amplitude and translocation dwell time (Figure 3ii). Despite partial overlap among the distributions, the signal amplitude exhibited a distinct upward shift with increasing gradient. This trend was confirmed by the peak positions of Gaussian‐fitted normalized amplitude distributions. The observed enhancement mainly arises from the reverse electrodialysis effect: a higher concentration gradient directly increases the driving force for ion migration, which in turn elevates the ion flux through the nanopores and enhances the current amplitude. Moreover, High‐concentration electrolytes significantly reduce the solution resistance, while charge fluctuations at the interface between the Ag/AgCl electrode and the electrolyte give rise to current‐independent 1/f‐type voltage noise. This noise is converted into prominent current noise via the low resistance, with the electrode noise parameters increasing approximately linearly with electrolyte concentration [32]. In the high‐concentration regime, this electrode‐derived noise becomes the dominant noise component. Concomitantly, the reduction in solution resistance leads to a slight elevation of thermal noise, which further contributes to the enhancement of total noise.

In contrast, Gaussian fitting of normalized translocation time distributions revealed no significant shift in the peak residence times under different gradient conditions. Although the absolute dwell times increased slightly, the effect was not visually discernible due to the millisecond time scale and the broad distribution range. The observed increase can be attributed to an asymmetric potential distribution across the nanopore. A higher resistance on the low‐concentration (cis) side results in a larger voltage drop, producing a stronger local electric field relative to the trans side. Meanwhile, the electroosmotic flow (EOF) acts opposite to the electrophoretic force on the analyte, jointly extending the translocation duration. However, as the residence times remain on the millisecond scale and the maximum concentration gradient is only tenfold, this effect is too small to shift the Gaussian peaks. Although a higher electrolyte concentration gradient improves signal amplitude and contrast, it also increases electrical noise and baseline fluctuations. Balancing these opposing effects, a trans‐side concentration of 0.1 m (no concentration gradient) was used in the following experiments to maintain signal stability and reproducibility.

#### 2.2.2 Driving Voltage

**FIGURE 4:**
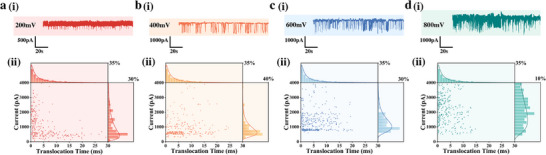
The influence of driving voltage on current and translocation time. (a) 200 mV, (b) 400 mV, (c) 600 mV, (d) 800 mV. (i) Typical current trace; (ii) Scatter diagram of event distribution and normalized fitting Gaussian distribution diagram.

To investigate the effect of driving voltage on the sensing performance of nanopores, experiments were conducted under applied voltages of 200 mV, 400 mV, 600 mV, and 800 mV. The electrolyte concentrations on both the cis and trans sides were maintained at 0.1 mol/L. The corresponding results are presented in Figure 4a–d. Both the frequency and amplitude of AFP‐induced current blockade events exhibited a gradual increase with the elevation of applied voltage. An increase in applied voltage expands the “effective capture radius” of the nanopore— the range of the electric field extends beyond the vicinity of the pore orifice. More molecules that were originally in diffusive motion and distant from the pore can be “trapped” at the pore entrance by the electric field, further enhancing the collision/binding probability between molecules and the nanopore. However, at 800 mV, significant baseline fluctuation and elevated electrical noise were observed, indicating the loss of signal stability under excessively high bias conditions. High voltage significantly boosts the migration velocity and flux density of ions within the nanopore. The randomness of ion collisions and diffusion is amplified, giving rise to additional ionic noise. Meanwhile, the translocation speed of target molecules is drastically accelerated under high voltage; the translocation time of some molecules through the pore may approach the temporal resolution of the detection system, leading to the misclassification of current blockade signals as noise. Additionally, conformational fluctuations of molecules under a strong electric field further increase the uncertainty of the detection signal.

The scatter plots of blockade amplitude versus dwell time (Figure 4ii) showed distinct amplitude clusters at each applied voltage. The mean amplitude increased from ∼300 pA at 200 mV to ∼500 pA and ∼1000 pA at 400 mV and 600 mV, respectively. At 800 mV, the amplitudes became widely dispersed, indicating reduced signal reproducibility. Gaussian fitting confirmed a consistent increase in peak amplitude with voltage, following a positive, non‐linear trend.

This phenomenon results from the combined influence of voltage on ion distribution and electric field distribution [33, 34, 35]. Increasing the bias accelerates ion migration, raises local ion concentration, and thereby reduces solution resistivity (ρ), which according to the cavity resistance relation $\left(R_{\mathrm{pore}}=\frac{\rho L}{\pi D^{2}}\right)$, enhances the current amplitude [36]. Higher voltages also concentrate field lines near the nanopore entrance, lowering the access resistance and further increasing the current. In addition, stronger transmembrane voltage amplifies the electrophoretic driving force, accelerating molecular translocation and improving signal intensity. Considering the trade‐off between signal enhancement and baseline stability, an applied voltage of 200 mV was chosen for subsequent experiments to ensure reliable and reproducible performance.

### 2.3 Influence of Different Parameters on Detection Efficiency

#### 2.3.1 Nanopore Quantity

Improving the detection efficiency of tumor markers is essential for enhancing sample throughput and enabling timely clinical decision‐making. In this study, the effects of nanopore array configuration and electrolyte concentration on the detection efficiency of the AFP antigen were systematically investigated. Detection efficiency was defined as the time from antigen introduction to the point where ionic current reached a stable, saturated level.

**FIGURE 5:**
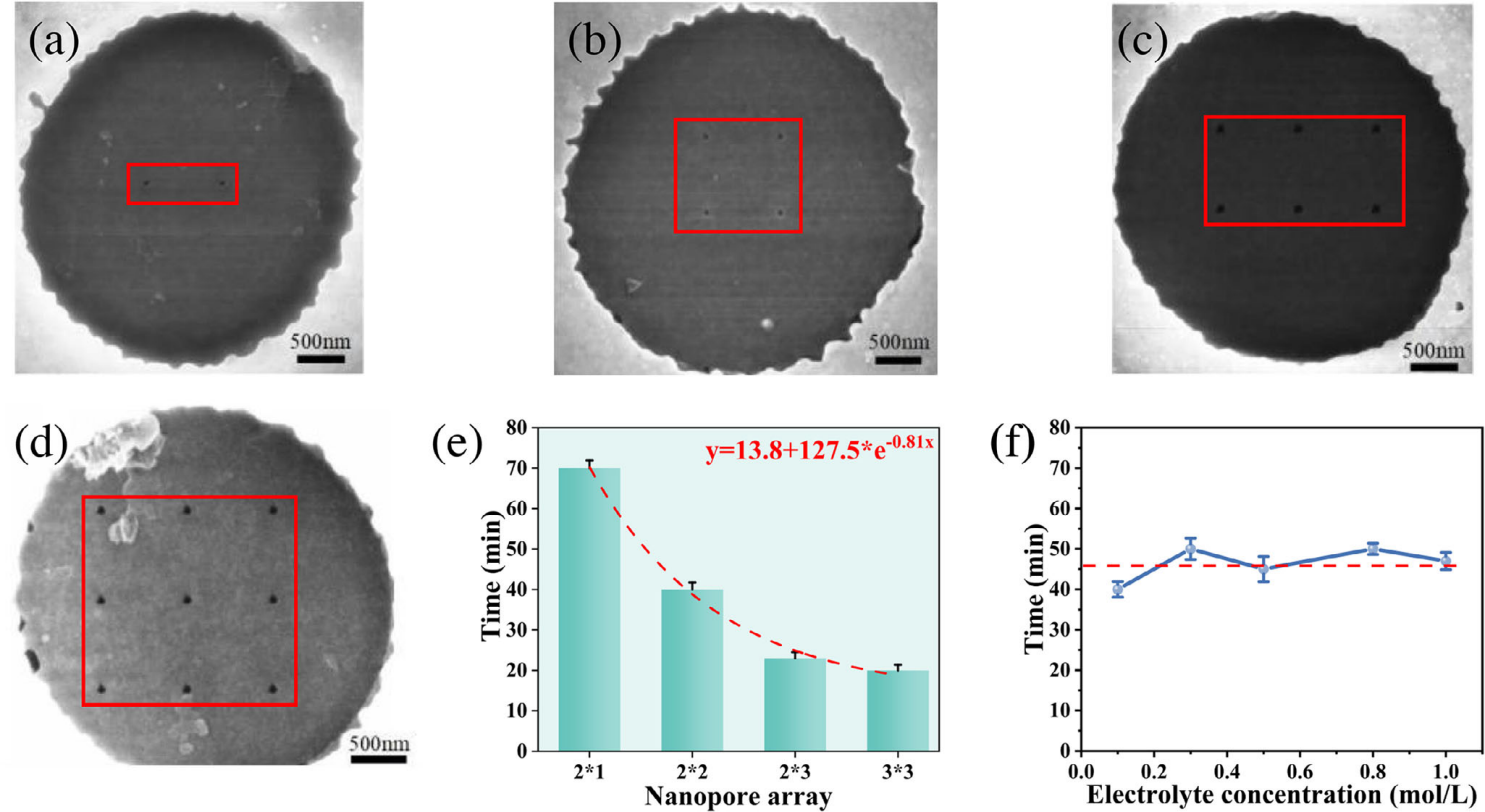
SEM diagram of Al_2_O_3_/Au/Si_3_N_4_ nanopore array. (a) A total of 2 × 1 nanopore array; (b) A total of 2 × 2 nanopore array; (c) A total of 2 × 3 nanopore array; (d) A total of 3 × 3 nanopore array. (e) Detection efficiency of different nanopore arrays; (f) Detection efficiency of 2 × 2 nanopore array under different electrolyte concentrations.

To investigate the regulatory effect of pore density on detection efficiency, nanopore arrays with four configurations (2 × 1, 2 × 2, 2 × 3, and 3 × 3) were examined, with the inter‐pore distance uniformly maintained at 800 nm (Figure 5a–d). As shown in Figure 5e, increasing the number of nanopores substantially reduced the detection time for the same antigen volume. Relative to the 2 × 1 array, detection times were reduced by 43%, 67%, and 71% for 2 × 2, 2 × 3, and 3 × 3 arrays, respectively. Fitting the data with an exponential decay model yields the equation: y = 16.5 + 131.8 × e^−0.44x^, which revealed that the detection time asymptotically approached a lower limit, indicating that the enhancement in efficiency gradually saturates with increasing pore number.

This saturation phenomenon arises from multiple interacting factors. An increased number of nanopores expands the total surface area for aptamer immobilization, enhancing binding site availability. It also enlarges the effective capture cross‐section, improving the likelihood of antigen binding via diffusion and electrophoresis. Moreover, when the inter‐pore distance exceeds the pore diameter, each nanopore functions independently, consistent with Poisson statistics, thereby facilitating earlier detection events. However, detection efficiency does not increase linearly with pore number. Diminishing returns are observed due to:

- amplified background noise from nanopores, the electrolyte environment, and stochastic ion translocation, reducing the signal‐to‐noise ratio;
- analyte depletion near the sensing region at low concentrations, which exacerbates diffusion limitations; and
- elevated pore densities that increase the risk of non‐specific adsorption and inter‐pore crosstalk, potentially introducing signal artifacts.

The results suggest that while increasing the number of nanopores enhances detection efficiency, the improvement eventually reaches a limit due to intrinsic transport constraints, finite antigen availability, and noise interference. This outcome provides theoretical guidance for the optimal design of nanopore sensors, highlighting the need to balance pore density with diminishing gains in detection performance

#### 2.3.2 Electrolyte Concentration

The influence of electrolyte concentration was evaluated by comparing the detection times for 10 fm AFP antigen using a 2 × 2 nanopore array under varying KCl concentrations (0.1 mol/L, 0.3 mol/L, 0.5 mol/L, 0.8 mol/L, and 1 mol/L). As shown in Figure 5f, the detection times across these concentrations were 40 min, 50 min, 45 min, 50 min, and 47 min, respectively, showing no significant trend.

The relatively stable detection times across electrolyte concentrations can be attributed to the nature of the aptamer‐antigen interaction, which relies primarily on Van Der Waals forces [37], rather than electrostatic attraction. Although the ionic strength of the electrolyte affects the number of counterions adsorbed onto the antigen surface, it has minimal influence on the specific binding affinity. Additionally, although antigen‐aptamer complexes carry net negative charges, leading to the formation of electric double layers within the nanopore. The Debye length of KCl within the electric double layer can be determined using the following formula: $\lambda_{D}=\sqrt{\frac{\varepsilon_{0}k_{B}T}{n_{e}e^{2}}}$. Where ε_0_ represents the vacuum permittivity (8.85 × 10^−12^ F/m). k_B_ denotes the Boltzmann constant (1.38 × 10^−23^ J/K). T corresponds to the temperature in Kelvin. The quantity n_e_ denotes the electron density per cubic meter, and e stands for the elementary charge (1.60 × 10^−19^ C).

The Debye length in 1 mol/L KCl (0.304 nm) is much smaller than the nanopore diameter. Thus, the surface potential's modulation of ionic current is negligible, and the electrolyte concentration has minimal effect on capture dynamics [38, 39]. These findings suggest that in the tested range, electrolyte concentration is not a dominant factor in determining the detection efficiency of the AFP antigen, and the system maintains robustness under varying ionic strengths.

### 2.4 Influence of Different Parameters on Equilibrium Dissociation Constant

#### 2.4.1 Concentration Gradient

**FIGURE 6:**
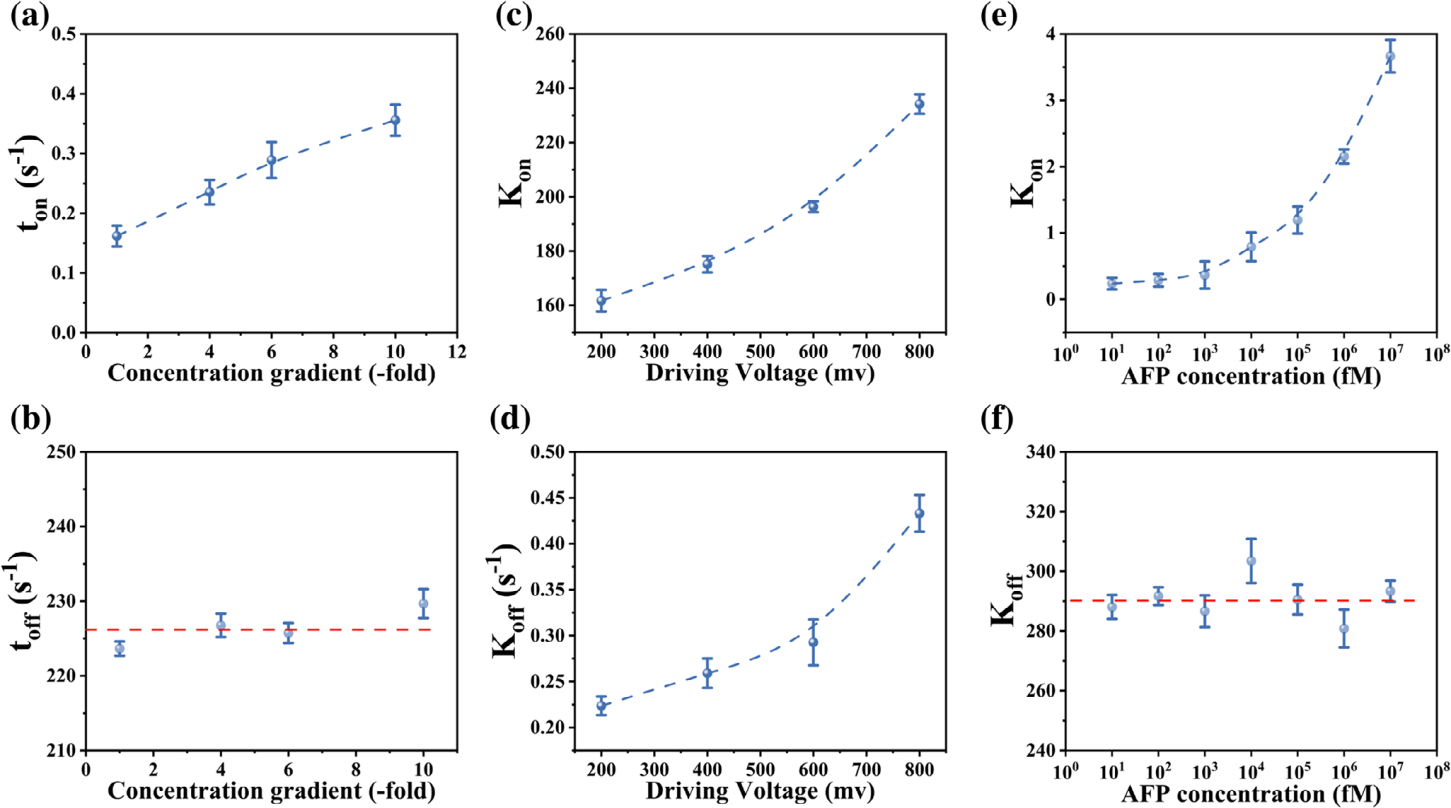
Influence of electrolyte concentration gradient on equilibrium dissociation constant. (a) t_on;_ (b) t_off._ Influence of different parameters on association rate constant (K_on_). (c) Driving voltage; (e) AFP concentration. Influence of different parameters on dissociation rate constant (K_off_). (d) Driving voltage; (f) AFP concentration.

The impact of trans‐side concentration gradients on the equilibrium dissociation constant was investigated by recording ionic current traces under 0.1 m, 0.4 m, 0.6 m, and 1.0 m conditions. Nanopore translocation signals were systematically filtered, and signals with d_well_ times longer than 1 ms were statistically analyzed. The resulting blockade signals(t_on_ and t_off_) were fitted using Gaussian models to extract peak values at each concentration gradient (Figure 6a,b).

To exclude the influence of protein concentration, the K_on_ was calculated using methods detailed in Supplementary T3. The values of K_on_ at increasing concentration were 0.162 s^−1^,0.235 s^−1^,0.289 s^−1^,0.345 s^−1^ respectively. These results reveal a monotonic increase in translocation frequency, reflecting shortened intervals between successive binding events. This trend is attributed to enhanced cation flux under steeper concentration gradients, which amplifies the local electric field and expands the nanopore's capture radius, thereby increasing analyte entry probability. Compared to baseline (0.1 m), K_on_ increased by 45.47%, 22.83%, and 19.63% at 0.4 m, 0.6 m, and 1.0 m, respectively, indicating a saturation effect at higher gradients.

In contrast, the K_off_ remained largely unchanged across all conditions, with values of 223.64 s^−1^,226.7574 s^−1^,225.73 s^−1^,229.67 s^−1^, showing no statistically significant variation. This indicates that the stability of the AFP–aptamer complex is not affected by gradient‐induced changes in the electric field. These findings confirm that the observed current blockades result from specific AFP–aptamer interactions within the nanopore sensing zone, rather than from non‐specific electrophoretic transport. The gradient‐dependent modulation of K_on_, combined with the stable K_off_, provides strong evidence for the active and specific binding of AFP molecules.

#### 2.4.2 Driving Voltage

The impact of transmembrane voltage on antigen–aptamer interactions was evaluated by recording ionic current traces under 200 mV, 400 mV, 600 mV, and 800 mV, with a fixed concentration gradient. Gaussian fitting of the current blockades yielded characteristic dwell times and amplitude values.

The K_on_ increased from 161.74 s^−^^1^ at 200 mV to 234.19 s^−^^1^ at 800 mV(Figure 6c), driven by the expansion of the nanopore capture radius and enhanced electrophoretic force on AFP molecules. Elevated voltage increases translocation velocity, resulting in more frequent binding events within a given time window [40, 41]. The K_off_ also increased from 0.22 s^−^^1^ at 200 mV to 0.43 s^−^^1^ at 800 mV (Figure 6d), indicating shorter complex d_well_ time under stronger electric fields. This trend reflects voltage‐induced mechanical destabilization of AFP–aptamer complexes. Fitting of unbinding time (t_off)_ revealed an exponential decay with voltage, consistent with prior single‐molecule force spectroscopy data [42].

These findings underscore the dual role of the applied voltage: it not only enhances molecular delivery into the nanopore sensing zone but also perturbs the energy landscape of binding interactions. Therefore, quantitative interpretation of equilibrium constants in voltage‐driven nanopore systems must account for both kinetic and mechanical contributions to molecular unbinding.

#### 2.4.3 AFP Concentration

The influence of AFP concentration on the equilibrium dissociation constant was examined using antigen solutions at 10 fm, 100 fm, 1 pm, 10 pm, 100 pm, and 1 nm. The blockade current signals(t_on_ and t_off_) were statistically analyzed, and Gaussian fitting was applied to determine peak values at each concentration. Based on these results, the K_on_and K_off_ were calculated according to Supplementary T3 (Figure 6e,f).

As shown in Figure 6e, K_on_ increased from 0.2379 s^−^^1^ at 10 fm to 3.666 s^−^^1^ at 1 nm. This monotonic increase reflects a shortened interval between successive blockade events, driven by the higher molecular flux of AFP at elevated concentrations. Greater antigen availability enhances encounter probability, accelerating binding frequency. In contrast, Figure 6f shows that K_off_ remained constant across all concentrations, averaging approximately 290 s^−1^. The consistency of dissociation kinetics suggests that binding duration is unaffected by antigen concentration, confirming that the observed current blockades result from specific AFP–aptamer interactions rather than nonspecific or voltage‐driven events.

### 2.5 Quantitative Detection of AFP Antigen via Nanopore Current Response

Quantitative assessment of the influence of the AFP antigen concentration on nanopore current was conducted by monitoring real‐time ionic current changes across a concentration range of 0.7 µm, 7 nm, 70 pm, 700 fm, and 70 fm, aiming to establish a robust detection framework. The total current drop (ΔI) was attributed to two distinct contributions: aptamer functionalization (ΔI_A_, Process A) and antigen binding (ΔI_B_, Process B), which exhibits a competitive relationship in signal generation. The current drop induced by aptamer modification (ΔI_A_) is constant, and since ΔI =ΔI_A_ +ΔI_B_, the magnitude of the ratio ΔI_A_/ΔI is inversely proportional to the current drop arising from antigen‐aptamer binding (ΔI_B_). Distinct antigen concentrations correspond to different current drops induced by antigen‐aptamer binding, thereby enabling the acquisition of unique ratios ΔI_A_/ΔI and ΔI_B_/ΔI for each concentration. By calculating the ratios ΔI_A_/ΔI and ΔI_B_/ΔI, we systematically analyzed the impact of antigen concentration on nanopore response and determined the sensor's detection limit.

**FIGURE 7:**
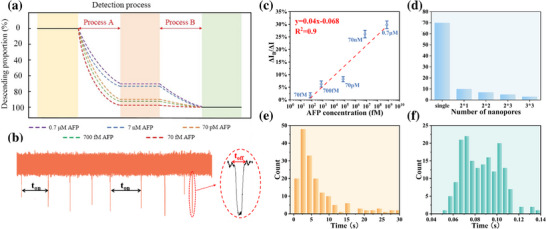
(a) The ratio of current drop of Al_2_O_3_/Au/Si_3_N_4_ single nanopore sandwich sensor to detect different concentrations of AFP antigen molecules; (b) Schematic diagram of t_on_ and t_off_ in current trace; (c) Linear fitting between ΔI_B_/ΔI and AFP concentration of single nanopore sensor; (d) LOD of different nanopore arrays;(e) Histogram of statistical distribution of t_on_; (f) Histogram of statistical distribution of t_off_.

As shown in Figure 7a, ΔI_B_/ΔI decreases with decreasing antigen concentration, whereas ΔI_A_/ΔI increases, indicating a transition from antigen‐binding‐dominated current blockade (Process B) to aptamer‐modification‐induced baseline suppression (Process A). At high antigen concentrations, AFP molecules rapidly bind to surface‐immobilized aptamers, leading to substantial ionic current reduction, with Process B being the dominant contributor. In contrast, at low concentrations, the probability of antigen‐aptamer binding events significantly decreases, reducing Process B's contribution while Process A remains unchanged, causing ΔI_A_/ΔI to rise. When the antigen concentration reaches 70 fm, ΔI_A_/ΔI approaches saturation (∼100%), marking the detection threshold where antigen binding no longer produces a discernible current drop.

A quantitative detection model was used to examine the correlation between ΔI_B_/ΔI and antigen concentration. As shown in Figure 7c, a clear logarithmic dependence is observed across the 70 fm to 0.7 µm range, reflecting the diminishing response at higher concentrations due to signal saturation. The red solid line represents a linear fit on the logarithmic scale, $f\left(\frac{\Delta I_{B}}{\Delta I}\right)=0.04\times \left(\mathrm{fm}\right)$ − 0.068, demonstrating strong correlation despite minor deviations, which are likely caused by heterogeneous surface functionalization, local nanopore environment variability, or limited sampling statistics.

Mechanistic insights arise from the interplay between aptamer modification (Process A) and specific antigen binding (Process B). At high antigen concentrations, specific binding dominates the current blockade signal. In contrast, at low concentrations, signal changes largely reflect baseline shifts from aptamer modification, marking the boundary where specific events become indistinguishable from background noise. Under these conditions, mass transport limitations outweigh translocation kinetics [43]. Target molecules must diffuse into the nanopore's capture zone before binding occurs, and at femtomolar levels, this probability is exceedingly low. As a result, both blockade frequency and amplitude decrease, constraining sensitivity.

To address this limitation and expand the dynamic range, strategies that enhance molecular transport are critical. These include salt gradients, dielectrophoretic forces, magnetic bead enrichment, and microfluidic flow control. Additional improvements may be achieved by increasing aptamer surface density, optimizing electric field distribution, and employing advanced signal amplification techniques.

### 2.6 Impact of Nanopore Numbers on AFP Detection Limit

A series of Al_2_O_3_/Au/Si_3_N_4_ sandwich‐structured sensors with single and arrayed nanopores (2 × 1, 2 × 2, 2 × 3, and 3 × 3 configurations) were fabricated to explore the relationship between nanopore number and LOD, maintaining a uniform inter‐pore distance of 800 nm. AFP‐specific aptamers were immobilized on the Au layer via Au─S bonds to enable selective target capture. All measurements were performed under constant conditions: 200 mV bias, 1 m KCl electrolyte, and 50 µg/mL aptamer concentration. Upon AFP introduction, electrophoretic transport drives the antigen toward the nanopore, where it binds specifically to the aptamer, partially obstructing ion flow and resulting in measurable current blockade. At sub‐threshold concentrations, however, current signals remain indistinguishable from baseline noise (Figure S7b), indicating insufficient binding events for detection. By titrating AFP concentrations and identifying the onset of distinguishable current deviations, LODs were determined as 70 fm (single pore), 10 fm (2 × 1), 7 fm (2 × 2), 5 fm (2 × 3), and 3 fm (3 × 3) (Figure 7d). Compared with existing AFP detection methods reporting LODs of 714 pm and 1.18 pm [44, 45], the 3 × 3 nanopore array demonstrates significantly improved sensitivity. This performance not only meets clinical diagnostic thresholds [46], but also highlights the scalability and optimization potential of array‐based nanopore sensing platforms.

The sensitivity of the nanopore‐based sensor exhibits a clear positive correlation with the number of integrated nanopores. This enhancement can be attributed to two synergistic factors: (i)expansion of the effective capture area elevates the local concentration of target molecules near the sensing zone, enhancing interaction likelihood [47]; (ii) a greater number of surface‐immobilized aptamers provides more recognition sites, increasing binding probability and amplifying ionic current signals [48]. As a result, increasing the number of nanopores improves detection performance by lowering the LOD, expanding the dynamic range, and enhancing measurement accuracy. However, these improvements diminish beyond a critical nanopore density, where inter‐pore interference, steric hindrance, and non‐uniform molecular diffusion lead to performance saturation. Excessive signal redundancy and accumulated noise further complicate data interpretation, reflecting the nonlinear and saturating trend of LOD reduction.

Beyond AFP detection, the aptamer–nanopore platform offers high modularity and chemical specificity, enabling adaptation to a wide range of clinically relevant cancer biomarkers. Integration with advanced signal amplification strategies, such as enzymatic enhancement, nanoparticle tagging, or digital thresholding [49, 50], further supports high‐throughput, multiplexed screening for clinical diagnostics and point‐of‐care applications.

### 2.7 Binding Affinity Characterization of AFP Aptamers Using Nanopore Arrays

A 2 × 3 Al_2_O_3_/Au/Si_3_N_4_ nanopore array functionalized with AFP‐specific aptamers was employed to investigate the binding affinity between the AFP and its aptamer. Upon introducing 10 fm AFP antigen into the cis chamber, the molecules were electrophoretically driven through the nanopores. A fraction of them specifically bound to aptamers immobilized on the nanopore inner walls, generating transient current blockades. Each current spike corresponds to a single binding event between an antigen molecule and the aptamer. These discrete blockade signals reflect the real‐time resolution of individual molecular interactions, enabling single‐molecule analysis of aptamer‐antigen binding kinetics.

Following initial capture, the system reached a dynamic equilibrium between binding and dissociation. Upon antigen unbinding, the current returned to baseline. The binding affinity was quantitatively characterized by the K_d_, calculated as 3.35 × 10^−10^ m from kinetic rate constants. A lower K_d_ indicates stronger binding, typically associated with more frequent events and longer dwell times, resulting in higher event density and improved signal‐to‐noise ratio. Statistical distributions to t_on_ and t_off_ are shown in Figure 7e,f, with summary values provided in Table S4 (event counts: N_1_ and N_2_)

The measured K_d_ value indicates a strong affinity. Comparatively, surface plasmon resonance (SPR) based assays by Huang et al. Reported aptamer‐AFP affinities in the low nanomolar range [51], while Shin et al. screened AFP_5 and AFP_12 via SELEX, with K_d_ values of 1.23 × 10^−^^9^ m and 2.52 × 10^−9^ m [52], respectively. These comparisons demonstrate that the aptamer employed in this work exhibits competitive or superior affinity, enabling stable antigen capture even at sub‐picomolar concentrations.

Variations in binding affinity across aptamers are primarily driven by sequence‐dependent folding patterns and selection conditions, including ionic strength, temperature, and target conformation. Environmental parameters—such as pH, salt concentration, and thermal stability — can modulate K_on_ and K_off_ by altering molecular dynamics [53, 54]. While nonspecific interactions may still occur in complex matrices, the current spikes observed under experimental conditions were sharp and well resolved (Figure 7b), indicating minimal background interference and high signal clarity. Beyond analytical performance, high‐affinity aptamers hold strong translational potential. Their low K_d_ values support early‐stage detection of biomarkers like AFP, essential for timely cancer diagnosis and intervention. In addition, their high specificity and real‐time responsiveness enable longitudinal disease monitoring, offering pathways toward personalized diagnostics and adaptive therapeutic strategies.

## 3 Conclusion

This work highlights a conceptual shift in solid‐state nanopore development by demonstrating that the integration of theoretical guidance, advanced nanofabrication, and selective biochemical functionalization strategies can effectively overcome long‐standing limitations in nanopore array design. By experimentally validating the impact of inter‐pore spacing on sensing performance and introducing a multilayer structural strategy that addresses both material and fabrication constraints, we provide a reproducible framework for designing high‐density nanopore systems with independent pore behavior.

This approach does not merely offer incremental improvements but establishes a paradigm for rational nanopore engineering. It enables the transition from empirical, low‐yield fabrication to predictable, scalable architectures with tunable sensing properties. The success of this platform opens up new possibilities for nanopore arrays in intelligent sensing and molecular analytics, where large‐scale, high‐resolution, and real‐time detection is required.

Looking forward, this strategy paves the way for integrating solid‐state nanopore arrays with on‐chip microelectronics, facilitating the creation of fully integrated, single‐molecule analytical systems. Moreover, coupling such platforms with AI‐powered signal interpretation could allow real‐time decoding of massive molecular event streams, enabling automated, high‐accuracy diagnostics. Together, the design principles and experimental breakthroughs reported here support the vision of solid‐state nanopores as foundational components in the next generation of analytical technologies—precise, intelligent, and ready for system‐level integration.

## 4 Experimental Section

### 4.1 Materials and Apparatus

Sulfuric acid solution (98%) and hydrogen peroxide solution (30%) were purchased from Zx‐Technology (Guangzhou). Anhydrous ethanol (99.5%) was supplied by Samhoo Trading Co., Ltd. (Guangzhou). KCl and PBS (pH 7.3‐7.5) were purchased from Aladdin. 3‐Aminopropyltriethoxysilane (98%) was provided by Wenrui Scientific Instrument Co., Ltd. (Guangzhou). Ethanolamine was obtained from Innochem (Beijing), and glutaraldehyde was procured from Wego (Guangzhou). α‐fetoprotein antigen and α‐fetoprotein aptamer were acquired from Sangon Biotech (Shanghai).

AFP aptamer sequence: 5'‐GTGACGCTCCTAACGCTGACTCAGGTGCAGTTCCGACTCGGTCTTGATGTGGGTCCTGTCCGTCCGAACCAATC ‐3' (5'modification: 5'SHC6; 3' modification: 3'6‐FAM).

AFP aptamer sample solution: Add an appropriate amount of AFP aptamer to 1 m PBS solution, then dilute it with different volumes of deionized water, and finally adjust the concentration to 50 µg/mL.

AFP antigen sample solution: Use a 0.1–2 µL pipette to take 1 µL of AFP antigen solution (1 mg/mL), add it to an appropriate volume of 1 m PBS solution, and then dilute it with different volumes of deionized water. Finally, prepare AFP antigen solutions with concentrations of 0.7 µm, 7 nm, 1 nm, 100 pm, 70 pm, 10 pm, 1 pm, 700 fm, 70 fm, and 10 fm, each with a volume of 10 mL.

The helium ion microscope (Orion NanoFab) and high‐resolution laser confocal microscope (LSM 800) were supplied by Carl Zeiss (Oberkochen, Germany), while the transmission electron microscope (Talos F200S) was purchased from Thermo Fisher Scientific (Waltham, USA). The ultra‐pure water machine (PURELAB Classic) was utilized to prepare deionized water for the experiment. An ultrasonic cleaning machine (YW0102) was employed to clean beakers, tanks, and gaskets. A thermostat water bath (HH2) was used to heat the chips, and a vacuum oven (DZF‐6050) was utilized to dry the chips. Axon Patch Clamp Equipment (AXON 700B) and a data acquisition system (Digidata 1550B) were employed to detect the ion current flowing through the nanopore, converting the analog current signal into a digital signal displayed on the computer screen.

### 4.2 Surface Functionalization

Figure S8 illustrates the schematic diagram of the AFP antigen detection principle using the modified Al_2_O_3_/Au/Si_3_N_4_ sandwich nanopores. Initially, the Al_2_O_3_/Au/Si_3_N_4_ sandwich nanopores were immersed in a mixed solution containing tris(2‐carboxyethyl)phosphine (TECP) and AFP aptamers with −SH groups. The AFP aptamers are covalently attached to the Au layer via gold–sulfur bonds, while the TECP solution reduces the disulfide bonds of the AFP aptamers, preventing their self‐aggregation (Step 2 in the diagram). Once the AFP aptamers are successfully immobilized on the Au surface of the nanopore walls, an external voltage is applied, which drives the negatively charged AFP antigens into the nanopores via electrophoresis. The specific binding between the AFP antigen and aptamer results in the capture of the antigen inside the nanopores (Step 3 in the diagram).

## Author Contributions

**Silu Feng**: Conceptualization, supervision, writing the original draft, and funding acquisition. **Qinglong Luo** and **Siqi Ai**: Conceptualization, data curation, and formal analysis. **Qinglong Luo**: Wring the original draft. **Suiwei Shen**: Data curation and formal analysis. **Chengyong Wang**: Supervision. **Zhishan Yuan**: Conceptualization, Supervision, data curation, formal analysis, and funding acquisition.

## Funding

This research was supported by Key‐Area Research and Development Program of Guangdong Province (No. 2023B0101200014), the National Natural Science Foundation of China (Nos. 52575480, 42577522, 52175388, and 32102078), and the Natural Science Foundation of Guangdong Province (No. 2021A1515012457), and Guangzhou Municipal Science and Technology (Project 2023A04J0351).

## Conflicts of Interest

The authors declare no conflicts of interest.

## Supporting information




**Supporting File**: advs74213‐sup‐0001‐SuppMat.docx.

## Data Availability

The data that support the findings of this study are available on request from the corresponding author. The data are not publicly available due to privacy or ethical restrictions.
