# Supplementary material for: Tri‐Layer Solid‐State Nanopore Arrays with Crosstalk Suppression for High‐Throughput, Femtomolar‐Level Biosensing
Source: Adv Sci (Weinh). 2026 Feb 4;13(20):e74213. doi: 10.1002/advs.74213 (PMC13067843; doi:10.1002/advs.74213)
Supplement: Supplementary file 1 — Supporting File: advs74213‐sup‐0001‐SuppMat.docx. [file ADVS-13-e74213-s001.docx]

Supporting Information

Tri-Layer Solid-State Nanopore Arrays with Crosstalk Suppression for High-Throughput, Femtomolar-Level Biosensing

*Silu Feng^*^, Qinglong Luo, Siqi Ai, Suiwei Shen, Chengyong Wang, Zhishan Yuan^*^*

**T1.** Nanopore Array Current Formula

The actual total current of the nanopore array is represented as I_1_, while the total current in the absence of inter-pore interference is denoted as I_lim_. The relationship between I and I_lim_ is given by the following equation:

$$\begin{aligned} \text{I}_{\text{1}}\text{=2πr}\text{z}_{\text{1}}\text{F}\text{D}_{\text{1}}\text{N}\int_{\text{0}}^{\text{r}} \frac{\text{∂}\text{c}_{\text{1}}\left( \text{x,m} \right)}{\text{∂y}}\text{|}_{\text{y=m}}\text{xdx} \end{aligned}\text{ (S1)}$$

$$\begin{aligned} \text{I}_{\text{lim}}\text{=4r}\text{z}_{\text{i}}\text{F}\text{D}_{\text{1}}\text{N}\text{c}_{\text{0}} \end{aligned}\text{ (S2)}$$

where F represents the Faraday constant, N is the number of nanopores, C_0_ is the initial concentration of cations in the electrolyte solution, D_1_ is the diffusion coefficient of the cations, and z_i_ is the valence of the cations in the electrolyte.

When the condition $\text{δ=}\text{I}_{\text{1}}\text{/}\text{I}_{\text{lim}}\text{≈}\text{1}$,which means that the inter-pore distance is greater than or equal to 20 times the nanopore radius ($\text{L}\text{≥}\text{20}\text{ }\text{r}$), diffusion zones do not overlap, allowing each nanopore to operate independently without interference. Based on this criterion, the inter-pore distances in Al₂O₃/Au/Si₃N₄ sandwich nanopore arrays designed for this study are set accordingly.

When the nanopore array consists of N nanopores, as shown in Figure 2(a), with each nanopore connected in parallel, the formula for the baseline current is given by:

$$\begin{aligned} \text{R}_{\text{total }\text{channel}}\text{= }\frac{\text{ρ}}{\text{N}}\left( \frac{\text{4l}}{\text{π}\text{d}^{\text{2}}} \right) \end{aligned}\text{(S3)}$$

$$\begin{aligned} \text{R}_{\text{total }\text{access}}\text{=ρ}\frac{\text{ϵ}}{\text{C}}\text{=}\frac{\text{ρ}}{\text{Nd}}\left( \text{1+}\text{γ}_{\text{n}}\frac{\text{d}}{\text{2L}} \right) \end{aligned}\text{(S4)}$$

$$\begin{aligned} \text{G=}\frac{\text{I}}{\text{U}}\text{=}\left( \text{R}_{\text{total }\text{channel}}\text{+}\text{R}_{\text{total }\text{access}} \right)^{\text{-1}}\text{=Nσ}\left[ \frac{\text{4l}}{\text{π}\text{d}^{\text{2}}}\text{+}\frac{\text{1}}{\text{d}_{\text{eff}}} \right]^{\text{-1}} \end{aligned}\text{(S5)}$$

$$\begin{aligned} \text{d}_{\text{eff}}\text{=d/(1+}\text{γ}_{\text{n}}\frac{\text{d}}{\text{2L}}\text{)} \end{aligned}\text{(S6)}$$

$$\begin{aligned} \text{I=U}\left( \text{R}_{\text{total }\text{channel}}\text{+}\text{R}_{\text{total }\text{access}} \right)^{\text{-1}}\text{=NσU}\left[ \frac{\text{4l}}{\text{π}\text{d}^{\text{2}}}\text{+}\frac{\text{1}}{\text{d}_{\text{eff}}} \right]^{\text{-1}} \end{aligned}\text{(S7)}$$

As illustrated by the nanopore array in Figure 2(a), when the nanopores are arranged in a two-dimensional configuration, $\text{γ}_{\text{n}}\text{\textasciitilde}\text{N}^{\text{1/2}}$.

**T2.** COMSOL Model and Parameters of the Nanopore Array

The 3D model (**Figure S9**), constructed to closely replicate the actual geometry of the nanopore array, couples the Electrostatics and Transport of Diluted Species physics interfaces with the Nernst–Planck and Poisson equations. This integrated approach enables detailed analysis of how inter-pore distance influences ion current and reveals position-dependent effects across the nanopore array. The nanopore array is 3×3, each with a diameter of 30 nm and a length of 15 nm, connected by two electrolyte-filled chambers. The inter-pore distance between adjacent nanopores is variable. Both the top and bottom electrolyte-filled chambers have radii and lengths of 1000 nm. The mesh size for the nanopore walls is set to 1.5 nm, which the mesh for the remaining geometries is automatically generated as tetrahedral mesh.

The physics interfaces for The physics interfaces for Electrostatics and Transport of Diluted Species are coupled with the Nernst-Planck equations and Poisson’ s equation, providing a comprehensive framework for simulating ion transport and electric field interactions within the nanopore array.

In the COMSOL model, the electrostatic potential (V) at the liquid chamber interfaces is set as V = 0 at the Cis side (layer A) and V = 200 mV at the Trans side (layer B), representing the application of Ag/AgCl electrodes at both ends of the electrolyte-filled chambers. The detailed simulation parameters and boundary conditions for the nanopore array COMSOL model are provided in Table S1.

In this model, variations in the inter-pore distance lead to differences in ion current across nanopores located at various positions within the array. To calculate the simulated ion current for each nanopore, the following formula is used:

$$\begin{aligned} \text{I}\text{=}\int\text{F}\left( \text{z}_{\text{K}_{\text{+}}}\text{N}_{\text{K}_{\text{+}}}\text{+}\text{z}_{\text{Cl}^{\text{-}}}\text{N}_{\text{Cl}^{\text{-}}} \right)\text{ndS}\text{=}\int_{\text{0}}^{\text{R}} \text{2}\text{πrF}\left( \text{z}_{\text{K}_{\text{+}}}\text{N}_{\text{K}_{\text{+}}}\text{+}\text{z}_{\text{Cl}^{\text{-}}}\text{N}_{\text{Cl}^{\text{-}}} \right)\text{d}\text{r} \end{aligned}\text{(S8)}$$

Where F is Faraday constant，$\text{z}_{\text{K}_{\text{+}}}$、$\text{z}_{\text{Cl}^{\text{-}}}$ represents the ionic compounds of ion $\text{K}^{\text{+}}\text{and }\text{Cl}^{\text{-}}$，$\text{N}_{\text{K}_{\text{+}}}$、$\text{N}_{\text{Cl}^{\text{-}}}$ denotes the ionic flux of ions $\text{K}^{\text{+}}\text{ and }\text{Cl}^{\text{-}}$ along the nanopore axis (z-axis), while S represents the cross-sectional area of the nanopore along the axis. R indicates the radius of the nanopore.

**T3.** Calculation formula of kinetic rate constant

The dissociation rate ($\text{K}_{\text{off}}$) reflects the rate at which aptamers and antigens dissociate from each other after forming a complex.The dissociation rate is calculated as follows

$$\begin{aligned} \text{K}_{\text{off}}\text{=}\frac{\text{1}}{\text{t}_{\text{off}}} \end{aligned}\text{(S9)}$$

where $\text{t}_{\text{off}}$ is the d_well_ time of the aptamer binding events. The association rate ($\text{K}_{\text{on}}$) characterizes the rate at which the aptamer and antigen form a complex.The association rate is calculated as follows

$$\begin{aligned} \text{K}_{\text{on}}\text{=}\frac{\text{1}}{\text{c•}\text{t}_{\text{on}}} \end{aligned}\text{(S10)}$$

where $\text{t}_{\text{on}}$ is the time in between aptamer binding events and c is the aptamer concentration. The dissociation constant $\text{(K}_{\text{d}}\text{)}$ is calculated as follows

$$\begin{aligned} \text{K}_{\text{d}}\text{=}\frac{\text{K}_{\text{off}}}{\text{K}_{\text{on}}} \end{aligned}\text{(S11)}$$

Based on equations (S9) and equations (S10), we calculated the binding rate constant ($\text{K}_{\text{on}}$) and dissociation rate constant ($\text{K}_{\text{off}}$) respectively.

**Table. S1** Boundary Conditions and parameters for Nanopores array COMSOL Model

| Plane | Poisson  （electrical potential） | Nernst-Planck  （motion of ions） |
| --- | --- | --- |
| A | constant potential  $\text{φ}\text{=0}$ | ion concentration  $\text{c}_{\text{i}}$ |
| B、C | no charge | no flux  $\text{n∙}\text{N}_{\text{i}}\text{=0}$ |
| E、F、H、I、J、L、M、N、O、P、Q | zero charge | no flux  $\text{n∙}\text{N}_{\text{i}}\text{=0}$ |
| D | constant potential  $\text{φ}\text{=}\text{V}$ | ion concentration  $\text{c}_{\text{i}}$ |

**Table. S2** Distance Variation Parameters for Adjacent Nanopores

| Group No. | 1 | 2 | 3 | 4 | 5 | 6 | 7 | 8 | 9 |
| --- | --- | --- | --- | --- | --- | --- | --- | --- | --- |
| Inter-pore distance $\text{L}$（nm） | 50 | 100 | 160 | 240 | 300 | 360 | 400 | 440 | 480 |
| Nanopore radius $\text{r}$（nm） | 15 | 15 | 15 | 15 | 15 | 15 | 15 | 15 | 15 |
| $\text{L}\text{/}\text{r}$ | 10/3 | 20/3 | 32/3 | 16 | 20 | 24 | 80/3 | 88/3 | 32 |

**Table. S3** Al_2_O_3_/Au/Si_3_N_4_ Nanopore Sandwich Processing Parameters

| ionic current（pA） | Ion dose（nC/um^2^） | | | | | | | | | |
| --- | --- | --- | --- | --- | --- | --- | --- | --- | --- | --- |
| 20pA | 50 | 70 | 90 | 110 | 130 | 180 | 230 | 280 | 330 | 380 |
| 15 pA | 50 | 70 | 90 | 110 | 130 | 180 | 230 | 280 | 330 | 380 |
| 10 pA | 50 | 70 | 90 | 110 | 130 | 180 | 230 | 280 | 330 | 380 |

**Table. S4** Summary of affinity data

| $\text{t}_{\text{on}}$ (s) | N_1_ | $\text{t}_{\text{off }}$(ms) | N_2_ | $\text{K}_{\text{d}}$ (nM) |
| --- | --- | --- | --- | --- |
| 5 ± 5 | 168 | 0.09 ± 0.03 | 144 | 0.1803 ± 0.1637 |


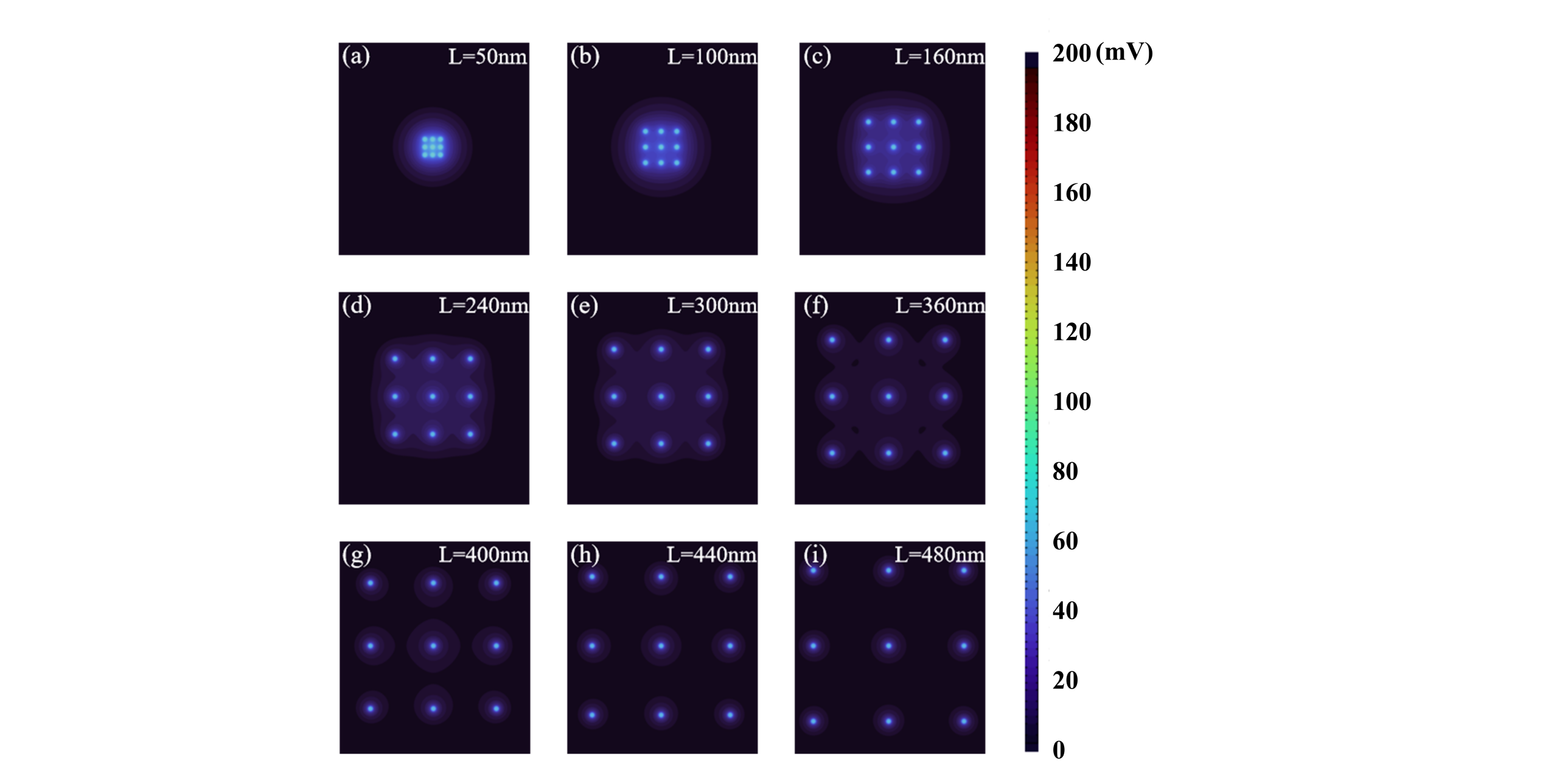
**Figure S1.** Potential profile of 3×3 nanopore array in xy plane at different inter-pore distance. **(a)** L=50nm; **(b)** L=100nm; **(c)** L=160nm; **(d)** L=240nm; **(e)** L=300nm; **(f)** L=360nm; **(g)** L=400nm; **(h)** L=440nm;

**(i)** L=480nm.


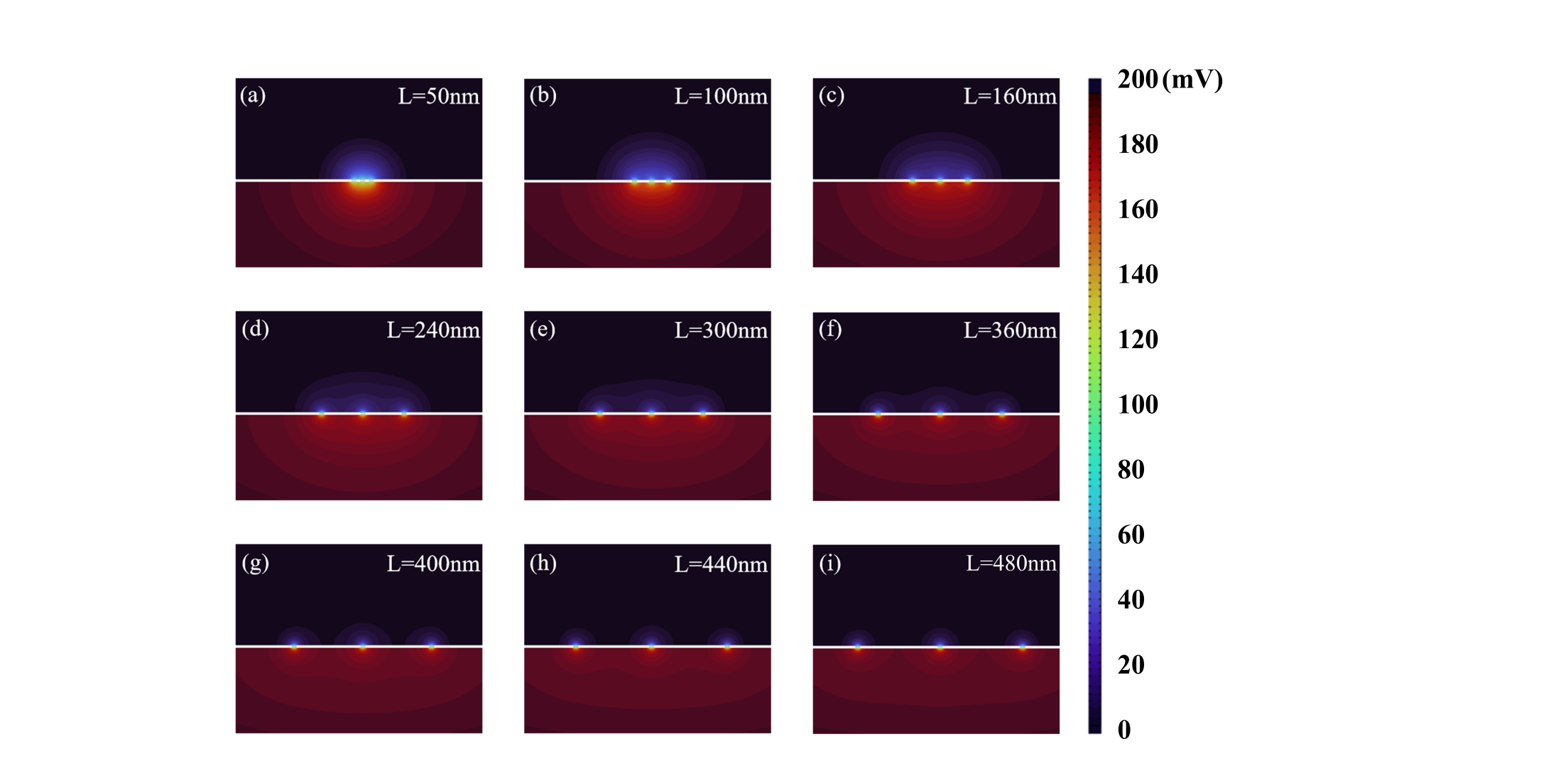


**Figure S2.** Potential profile of 3×3 nanopore array in yz plane at different inter-pore distance.

**(a)** L=50nm; **(b)** L=100nm; **(c)** L=160nm; **(d)** L=240nm; **(e)** L=300nm; **(f)** L=360nm; **(g)** L=400nm;

**(h)** L=440nm; **(i)** L=480nm.





**Figure S3.** Effect of electrolyte on current at different positions under different inter-pore distance.

**(a)** 50nm; **(b)** 100nm; **(c)** 160nm; **(d)** 240nm; **(e)** 300nm; **(f)** 360nm; **(g)** 400nm;**(h)** 440nm; **(i )**480nm.





**Figure S4.** Schematic diagram of the fabrication process flow of Al_2_O_3_/Au/Si_3_N_4_ membrane.


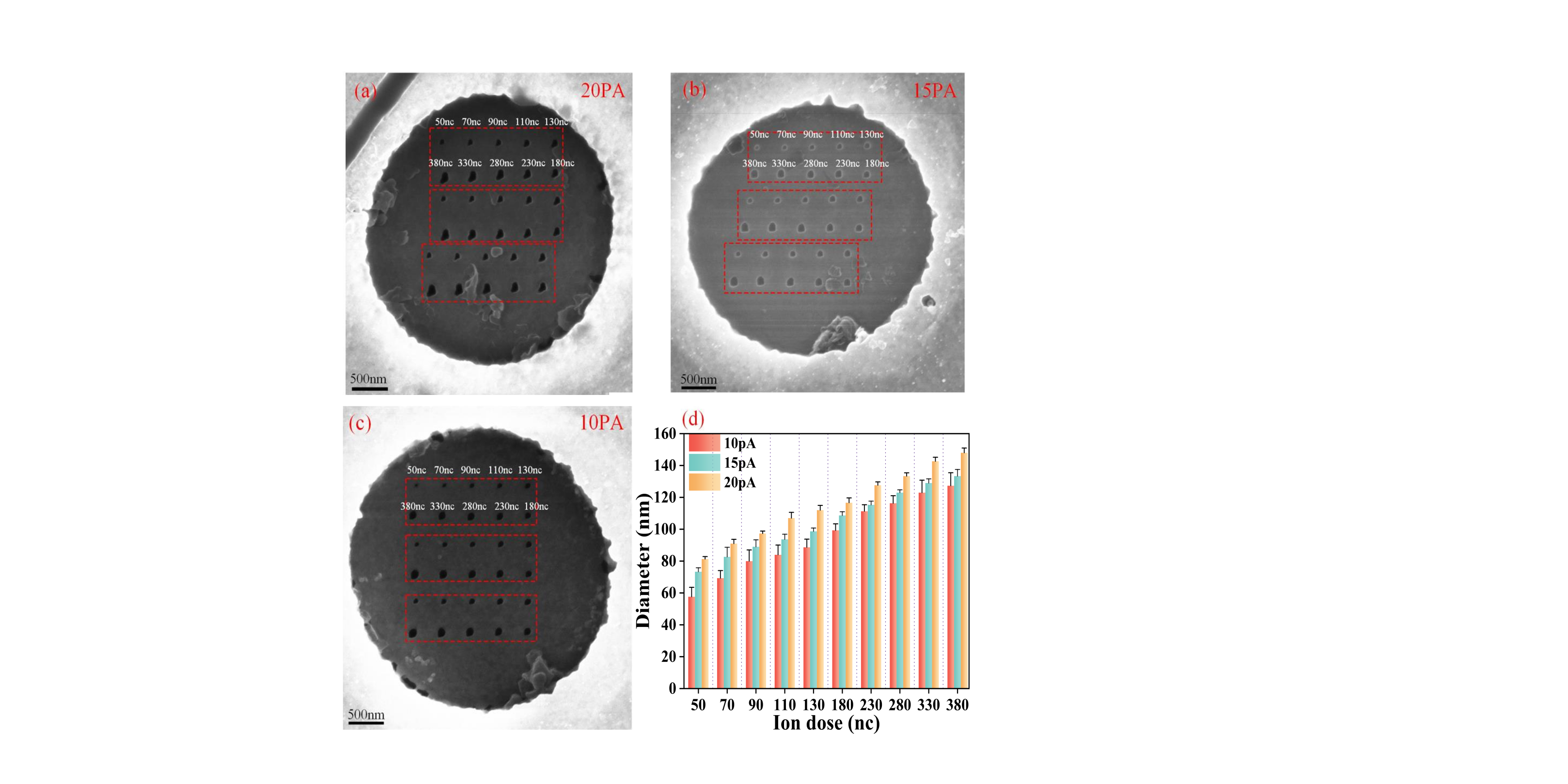


**Figure S5.** Al₂O₃/Au/Si₃N₄ sandwich structured nanopores fabricated with different processing parameters: **(a)** 20nA; **(b)** 15nA; **(c)** 10nA. **(d)** Bar graph illustrating the dependence of nanopore diameter on ion dose under current regimes of 10 pA, 15 pA, and 20 pA.


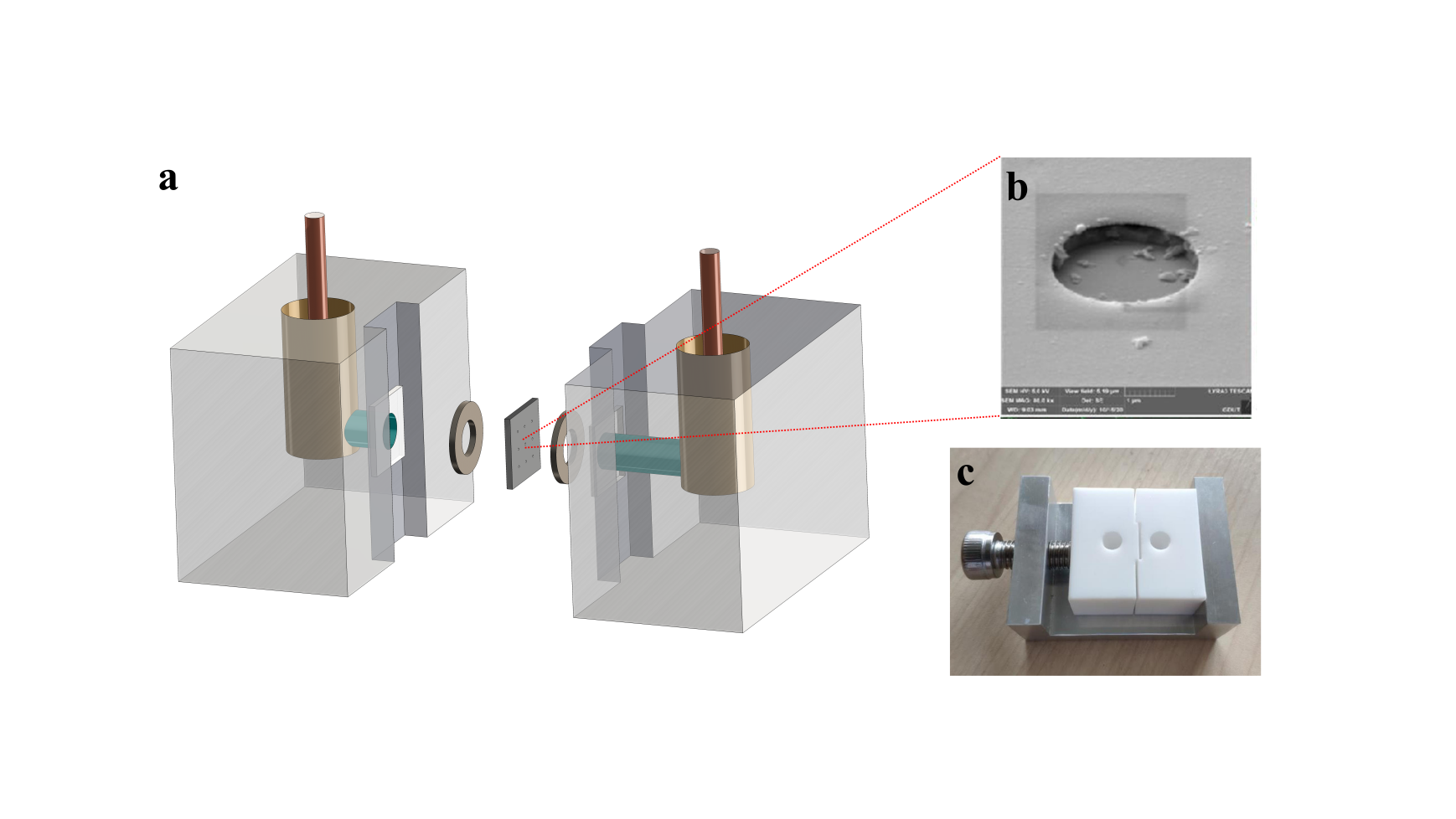


**Figure S6.** **(a)** Schematic diagram of chip clamping device. **(b)** SEM image of nanopore. **(c)** Physical image of chip clamping device.


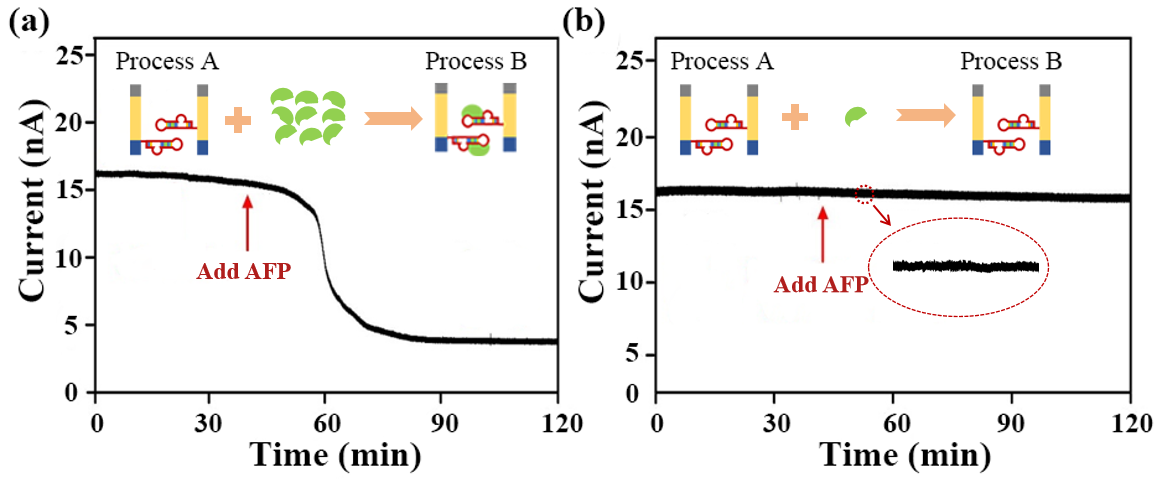


**Figure S7.** Current variation upon the addition of AFP into nanopores after surface functionalization.

**(a)** High concentration. **(b)** Low concentration.


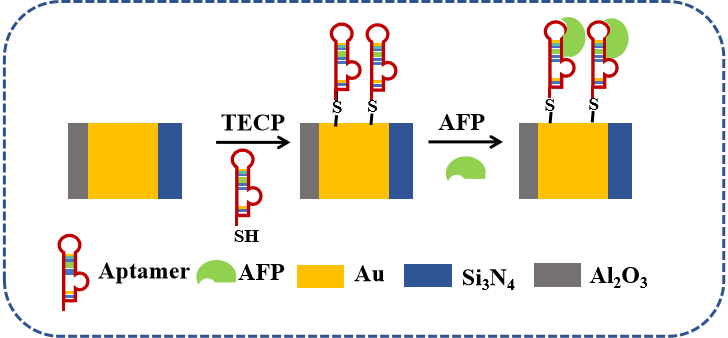


**Figure S8.** The modification scheme of Al_2_O_3_/Au/Si_3_N_4_ nanopores sandwich.


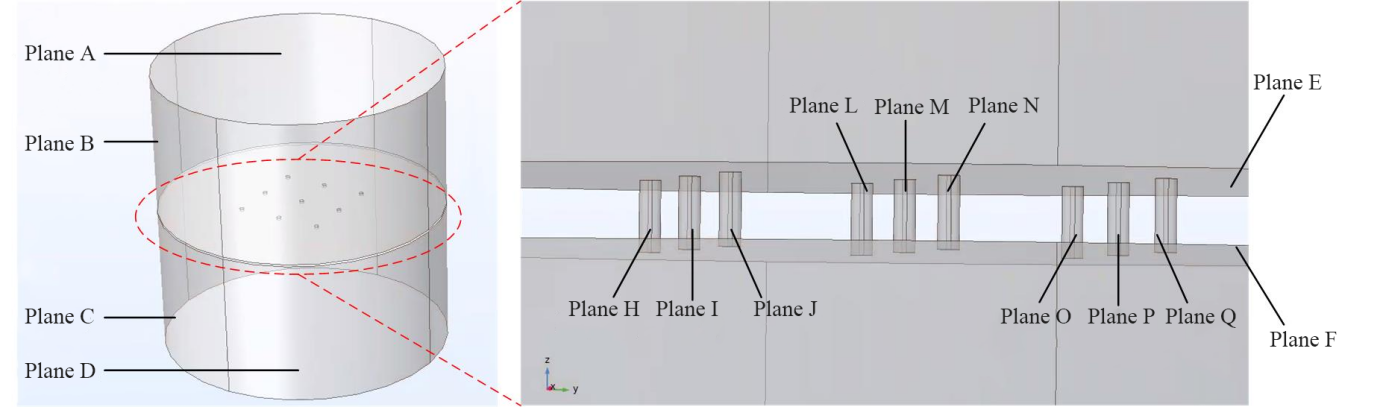


**Figure S9.** Schematic diagram of COMSOL simulation model of 3×3 array nanopores.
